# Acacetin Attenuates Lysophosphatidylcholine-Induced Vascular Smooth Muscle Cell Injury via Sirt1-Nrf2/p62 Signaling Axis

**DOI:** 10.3390/biomedicines14010194

**Published:** 2026-01-15

**Authors:** Yun-Da Li, Yao Wu, Tian-Li Zhou, Qian Yuan, Gui-Rong Li, Wei-Yin Wu, Yan Wang, Gang Li

**Affiliations:** 1 Xiamen Cardiovascular Hospital of Xiamen University, School of Medicine, Xiamen University, Xiamen 361000, China; yunda.li@xmu.edu.cn (Y.-D.L.); isabel_wuyao@xmu.edu.cn (Y.W.); drzhoutianli@outlook.com (T.-L.Z.); riley19930407@gmail.com (Q.Y.); grli8@outlook.com (G.-R.L.); wy@medmail.com.cn (Y.W.); 2 Nanjing Amazigh Pharma Limited, Nanjing 210032, China

**Keywords:** acacetin, lysophosphatidylcholines, atherosclerosis, vascular smooth muscle cells, Nrf2, Sirt1

## Abstract

**Background:** Acacetin, a naturally occurring flavone present in various plants, is known as a promising drug candidate for cardiovascular disorders. Our previous study demonstrated that acacetin ameliorates atherosclerosis through endothelial cell protection; however, its pharmacological effects on vascular smooth muscle cells (VSMCs) remain unexplored. This study investigates the therapeutic potential of acacetin against lysophosphatidylcholine (LysoPC)-induced VSMC injury and elucidates the underlying molecular mechanisms. **Methods and Results:** Multiple biochemical techniques were employed in the present study. The results showed that acacetin significantly attenuated LysoPC-induced apoptosis and reactive oxygen species (ROS) generation in cultured VSMCs. Western blot analysis revealed that the cytoprotection of acacetin was associated with upregulated expression of antioxidant defense proteins, including nuclear factor erythroid 2-related factor 2 (Nrf2), catalase (CAT), NADPH quinone oxidoreductase 1 (NQO-1), and superoxide dismutase 1 (SOD1). Nrf2 silencing completely abolished these protective effects. Mechanistically, siRNA-silencing of Sirtuin 1 (Sirt1) abrogated acacetin-induced modulation of the Nrf2/Keap1/p62 signaling. In vivo validation using aortic tissues from high-fat-diet-fed ApoE^−/−^ mice confirmed that acacetin effectively suppressed VSMC apoptosis and ROS overproduction associated with restoring the downregulated Sirt1 expression levels. **Conclusions:** These findings establish a novel mechanistic paradigm wherein acacetin confers protection against LysoPC-induced VSMC apoptosis and oxidative stress through Sirt1-dependent activation of the Nrf2/p62 signaling pathway, suggesting that acacetin is a promising therapeutic drug candidate for atherosclerotic plaque stabilization.

## 1. Introduction

Atherosclerosis (AS) is a vascular disorder characterized by dyslipidemia and chronic inflammation. Lysophosphatidylcholine (LysoPC), as the principal bioactive lipid component of oxidized low-density lipoprotein (ox-LDL), is significantly elevated in both plaque tissues and plasma of patients with atherosclerosis [1,2]. High plasma levels of LysoPC are positively correlated with plaque vulnerability and increased risk of adverse cardiovascular events [3]. LysoPC can directly act on VSMCs, inducing phenotypic switching, abnormal proliferation and migration, enhanced oxidative stress and inflammatory responses, and triggering various forms of programmed cell death [4,5,6]. These pathological alterations are closely associated with plaque instability, progression of vascular stenosis, and acute cardiovascular events in clinical settings [7,8].

Experimental evidence demonstrates that LysoPC may induce an elevation of cytosolic free Ca^2+^ in human coronary artery SMCs, rabbit coronary artery SMCs, and human umbilical vein endothelial cells [9,10,11]. Our prior mechanistic investigations further identified transient receptor potential canonical (TRPC) channels TRPC1 and TRPC3 as essential mediators of LysoPC-induced Ca^2+^ influx and subsequent apoptosis in human coronary artery SMCs. Genetic silencing of either TRPC isoform effectively abrogated LysoPC-triggered calcium overload and apoptotic signaling [12]. Despite these well-characterized pathogenic contributions, a critical translational gap persists: no currently available therapeutic agents specifically target LysoPC-mediated pathways to mitigate atherosclerotic progression. This unmet clinical need underscores the urgency for developing novel pharmacological strategies targeting LysoPC-associated molecular mechanisms.

Acacetin is a natural flavone compound ubiquitously present in *Robinia pseudoacacia* L., *Pseudostellaria heterophylla*, *Chrysanthemum morifolium*, and *Pogostemon cablin* [13,14] and exhibits a multifunctional pharmacological profile encompassing antimicrobial, anti-inflammatory, antioxidant, and antineoplastic properties [15,16,17,18,19,20], and diverse cardiovascular protective effects [14]. Beyond its well-documented atrial potassium channel blockade efficacy and atrial-selective atrial fibrillation [21,22,23,24], the cardio-protective potential of acacetin across diverse cardiovascular pathologies includes myocardial ischemia/reperfusion injury [25], cardiomyocyte hypoxia/reoxygenation damage [26], doxorubicin-induced cardiomyopathy and heart failure [27], pressure overload-induced cardiac hypertrophy [28], diabetic cardiomyopathy [29], and D-galactose-induced senescence-associated cardiomyopathy/heart failure [30]. Particularly relevant to vascular pathophysiology, acacetin demonstrates anti-atherosclerotic effects through endothelial protection mechanisms by activating Sirt1/Sirt3/AMPK signals under hyperglycemic conditions [31] and by activating the MsrA-Nrf2 pathway under Ox-LDL exposure [32]; however, the precise molecular mechanism behind the effects of acacetin on VSMCs, particularly under the specific stress with LysoPC, remains elusive. Although Nrf2 is the master regulator of antioxidant response, its canonical activation involves dissociation from Keap1. Recent evidence suggests a “non-canonical” pathway where the autophagy adaptor protein p62 (SQSTM1), upon phosphorylation, competes with Nrf2 for Keap1 binding. Whether acacetin is involved in the Sirt1/Nrf2/p62 axis to counteract LysoPC-induced vascular toxicity has not been investigated. To address this knowledge gap, the present study systematically evaluated the protective efficacy of acacetin against LysoPC-induced injury in rat aortic VSMCs and in aortic tissues of high-fat-diet-fed ApoE^−/−^ mice, employing a multidisciplinary experimental approach combining molecular biology and biochemical techniques.

## 2. Materials and Methods

### 2.1. Reagents and Antibodies

Acacetin (5,7-dihydroxy-4′-methoxyflavone) was chemically synthesized following established protocols with ≥99% purity [21] and water-soluble acacetin prodrug with 99.1% purity [22]. The in vitro concentrations 0.3–3 µM of acacetin and the in vivo dose 15 mg/kg/day (s.c.) of water-soluble acacetin prodrug used in this study were referred from the published literature [33].

The reagents including LysoPC and 2′,7′-dichlorofluorescein diacetate (DCFH-DA) were obtained from Sigma-Aldrich (St. Louis, MO, USA). Cell culture components comprising Dulbecco’s Modified Eagle Medium (DMEM), fetal bovine serum (FBS), 0.25% trypsin-EDTA, and antibiotic solutions (100 U/mL penicillin + 100 μg/mL streptomycin) were purchased from Thermo Fisher Scientific (Waltham, MA, USA), along with transfection reagents (Lipofectamine RNAiMAX, Opti-MEM) and calcium indicator Fluo-4 AM. Apoptosis detection was performed using an Annexin V-FITC/PI dual-staining kit from Dojindo Molecular Technologies (Kumamoto, Japan). Primary antibodies targeting Bcl-2 (ab32124), Bax (ab32503), Sirt1 (ab110304), Nrf2 (ab62352), Keap1 (ab119403), phospho-SQSTM1/p62 (Ser349, ab211324), catalase (CAT, ab16731), and SOD1 (ab13498) were procured from Abcam (Cambridge, UK), with additional antibodies against β-actin (sc-47778), Lamin B1 (sc-374015), NQO-1 (sc-32793), and p62/SQSTM1 (18420-1-AP) obtained from Santa Cruz Biotechnology (Dallas, TX, USA) and Proteintech (Rosemont, IL, USA), respectively. Gene silencing experiments utilized validated siRNAs targeting Nrf2 (sc-156128), Sirt1 (sc-108043), and non-targeting control (sc-37007) from Santa Cruz Biotechnology.

### 2.2. Cell Cultures

Primary vascular smooth muscle cells (VSMCs) were enzymatically isolated from thoracic aortic tissues of male Sprague-Dawley rats (200–250 g) following an established enzymatic digestion protocol [34]. Following isolation, cells were maintained in complete growth medium consisting of DMEM supplemented with 10% FBS, 100 U/mL penicillin, and 100 μg/mL streptomycin, under standardized culture conditions (37 °C, 5% CO_2_, 95% humidity). To ensure phenotypic stability and experimental consistency, all functional assays were conducted using cells between passages 3 and 8, corresponding to optimal viability and contractile marker preservation.

### 2.3. Cell Viability

VSMC viability was quantified using the MTT reduction assay according to standardized protocols [35]. Briefly, cells seeded in 96-well plates (5 × 10^3^ cells/well) were allowed to adhere for 12 h before pharmacological interventions. Cells were pretreated with pharmacologically relevant concentrations of acacetin (0.3, 1, and 3 μM)—dosages previously validated in cardiovascular models [26,27,28,32] or equivalent volumes of vehicle control (0.1% DMSO). Following 24 h co-incubation with LysoPC (30 μM) in low-serum DMEM (2% FBS), the MTT working solution (5 mg/mL in PBS, 100 μL/well) was introduced for 4 h at 37 °C. After careful aspiration of supernatants, formazan crystals were solubilized with anhydrous DMSO (150 μL/well) under gentle shaking. Optical density was measured at 570 nm using a microplate reader (BioTek Synergy H1, Agilent, Santa Clara, CA, USA), with viability values normalized to vehicle-treated control groups.

### 2.4. Aortic Tissue Acquisition and Experimental Design

The aortic root specimens analyzed in this study were derived from our established atherosclerosis model as previously reported [32]. Male ApoE^−/−^ mice (8-week-old, 22–25 g) were procured from Vital River Laboratory Animal Technology Co., Ltd. (Beijing, China) and maintained under specific pathogen-free conditions at Xiamen University Laboratory Animal Center (XMULAC). The experimental protocols were approved by the Ethic Committee for Animal Care and Use of Xiamen University (Approval No. XMULAC2020-0123). Following a 1-week acclimatization, mice were randomly stratified into three experimental cohorts: (1) Control group: Standard chow diet, (2) HFD group: Western diet (40% kcal fat, 1.25% cholesterol) for 12 weeks, and (3) HFD + Acacetin group: Western diet with concurrent subcutaneous acacetin administration (15 mg/kg/day) for 12 weeks. The therapeutic regimen was initiated synchronously with dietary induction to ensure temporal alignment of metabolic and pharmacological interventions. Acacetin was dissolved in vehicle solution (5% DMSO + 30% PEG300 + 65% saline) and administered via daily subcutaneous injection, while control groups received equivalent volumes of vehicle.

### 2.5. Cellular Apoptosis Quantification

Cellular apoptosis was assessed using dual-parameter flow cytometry with the Annexin V-FITC/PI Apoptosis Detection Kit (Dojindo, CK3001). For in vitro analysis, VSMCs seeded in 6-well plates (2 × 10^5^ cells/well) underwent pretreatment with acacetin (0.3, 1, 3 μM) or vehicle (0.1% DMSO in PBS) for 2 h prior to 24 h LysoPC (30 μM) challenge in 2% FBS DMEM. Following EDTA-free trypsinization (0.25% trypsin-EDTA, Thermo Fisher) and PBS (pH 7.4) washing, cells were re-suspended in 100 μL Annexin-binding buffer containing FITC-conjugated Annexin V (5 μL) and propidium iodide (5 μL) for 15 min dark incubation at RT. Flow cytometry analysis (Beckman Coulter Gallios, Brea, CA, USA) was performed within 1 h post-staining, with apoptotic populations quantified using Kaluza Analysis 2.1 software.

### 2.6. Plaque Apoptosis Analysis

In situ apoptosis within atherosclerotic plaques was evaluated through triple-i immunofluorescence-labeling using TUNEL/α-SMA/DAPI. Frozen aortic root sections (5 μm) underwent proteinase K digestion (20 μg/mL, 15 min) followed by TUNEL reaction mix (Beyotime, C1086) incubation (37 °C, 1 h). Sections were then blocked with 5% BSA and co-stained with α-SMA antibody (1:200, ab7817, Abcam) for 2 h at room temperature, followed by Alexa Fluor 594-conjugated secondary antibody (1:500, 1 h). Nuclear counterstaining with DAPI (1 μg/mL, 5 min) preceded mounting in anti-fade medium. Fluorescent images were acquired using the EVOS M7000 imaging system (Thermo Fisher) with consistent exposure settings across samples, and TUNEL^+^ α-SMA^+^ cells were quantified via ImageJ (v1.53) using threshold-based particle analysis.

### 2.7. Intracellular Calcium Flux Quantification

Dynamic intracellular free calcium ([Ca^2+^]_i_) fluctuations in VSMCs were monitored using real-time confocal imaging (Leica TCS SP5 II, Wetzlar, Germany) following established protocols [36,37]. Cells seeded in 35 mm-Dishes (Ibidi, 80136) at 2 × 10^4^ cells/cm^2^ density were serum-starved for 4 h prior to pretreatment with acacetin (0.3–3 μM) or vehicle (0.01% DMSO) in complete DMEM. Following pharmacological intervention, cells were loaded with 1 μM Fluo-4 AM (Thermo Fisher, F14201) in HEPES-buffered serum-free DMEM (pH 7.4) for 30 min at 37 °C under 5% CO_2_ humidified atmosphere. Post-incubation, three sequential washes with pre-warmed (37 °C) Tyrode’s solution (in mM: 140 NaCl, 5 KCl, 1 MgCl_2_, 2 CaCl_2_, 10 HEPES, 10 glucose, pH 7.4) were performed to remove extracellular dye. Time-lapse imaging was conducted at 23–25 °C using 488 nm excitation/515–530 nm emission settings, with images acquired at 2 s intervals over 300 s. Fluorescence intensity quantification was performed using Leica LAS X software (v3.7.4), with data normalized to baseline F_0_ values.

### 2.8. Reactive Oxygen Species (ROS) Quantification

Cellular ROS generation was assessed using DCFH-DA following established protocols [26]. VSMCs seeded in 6-well plates (1 × 10^6^ cells/well) were pretreated with acacetin (0.3, 1, 3 μM) or vehicle (0.05% DMSO) for 2 h prior to LysoPC (30 μM) stimulation in 2% FBS DMEM. For siRNA-mediated gene silencing experiments, cells transfected with Nrf2-targeting siRNA (50 nM) or scramble control (Santa Cruz, sc-37007) using Lipofectamine RNAiMAX were cultured for 48 h for protein knockdown before 24 h acacetin (3 μM) treatment. Following treatments, cells were loaded with 10 μM DCFH-DA in serum-free DMEM (37 °C, 30 min, dark), washed twice with Hanks’ Balanced Salt Solution (HBSS), and immediately analyzed by flow cytometry (Beckman Coulter Gallios, excitation/emission: 488/525 nm). A minimum of 10,000 events per sample were acquired, with fluorescence intensity quantified using Kaluza Analysis 2.1 (Beckman Coulter).

### 2.9. Vascular Oxidative Stress Assessment

In situ ROS production in atherosclerotic plaques was evaluated through dihydroethidium (DHE) fluorescence imaging. Aortic roots embedded in optimal cutting temperature (OCT) compound (Sakura, 4583, Tokyo, Japan) were cryosectioned into 5 μm thick slices using a CM1950 cryostat (Leica, Wetzlar, Germany). Sections were equilibrated to RT, incubated with 10 μM DHE (Sigma-Aldrich, D7008) in PBS (37 °C, 20 min, dark), and counterstained with DAPI (1 μg/mL, 5 min). After three PBS washes (5 min each), sections were mounted with ProLong Diamond Antifade (Thermo Fisher, P36961) and imaged under consistent parameters (40× objective, 561 nm excitation/610 nm emission) using the EVOS M7000 imaging system. DHE fluorescence intensity (≥5 fields/sample) was quantified using Image J (v1.53) with standardized threshold settings.

### 2.10. Nucleoprotein Extraction

The nuclear protein of Nrf2 was isolated using a Nuclear and Cytoplasmic Protein Extraction Kit (Beyotime, Beijing, China) in accordance with the manufacturer’s instructions. Unless stated, all steps were performed at 4 °C or on ice.

### 2.11. siRNA Transfection

Transfection of cells with siRNA was performed using Lipofectamine RNAiMAX transfection reagent, according to the manufacturer’s instructions. VSMCs at 60~70% confluence were transfected with siRNA molecules targeting rat Nrf2, Sirt1, and negative control at 100 nM. After transfection for 48 h, cells were used for determining ROS generation, protein expression, and cell apoptosis assays.

### 2.12. Western Blot Analysis

Western blot was performed as described previously [36]. Briefly, cells were lysed with a modified RIPA buffer (Solarbio, Beijing, China) containing 1% protease and phosphatase inhibitors (Roche, Germany). The protein concentration was determined using the BCA Protein Assay Kit (Thermo Fisher Scientific, Carlsbad, CA, USA). Cell lysates were mixed with sample buffer (5× loading buffer) and denatured by heating to 95 °C for 5 min. An equal amount of protein for each sample was resolved via 10% or 12% SDS-PAGE gel and then transferred to immunoblot PVDF membranes (Bio-Rad, Hercules, CA, USA). Membranes were blocked with 5% non-fat milk in Tris buffer saline with 0.1% tween-20 (TBST) for 1h, followed by overnight incubation at 4 °C with specific primary antibodies (at a range from 1:1000 to 1:2000). After being washed with TBST three times, the membranes were incubated with HRP-conjugated secondary antibodies at 1:10000 dilutions in TBST at room temperature for 1 h. Membranes were rewashed with TBST and then visualized with ECL reagents (Advansta, Menlo Park, CA, USA). The signals were detected with a chemiluminescence detection system (FluoChem E, Biotechne, Minneapolis, MN, USA). The relative band intensities were quantified by Image J software (NIH, Bethesda, MD, USA).

### 2.13. Statistical Analysis

Statistical analysis was performed using GraphPad Prism 9 (GraphPad Software, Inc., San Diego, CA, USA). Results are presented as means ± SEM. One-way ANOVA followed by post hoc Tukey’s test was used for comparison among groups. A value of *p* < 0.05 was considered statistically significant.

## 3. Results

### 3.1. Acacetin Mitigates LysoPC-Induced VSMC Apoptosis Through Bcl-2/Bax Regulation

**Figure 1:**
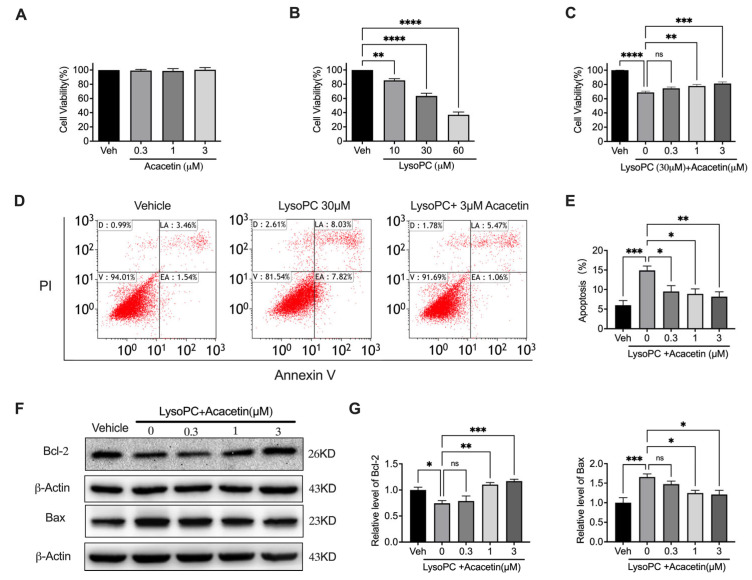
Acacetin suppressed LysoPC-induced apoptosis in VSMCs. (**A**) Cell viability was determined by MTT assay in VSMCs incubated with vehicle (Veh) or acacetin for 24 h (*n* = 6 in each group). (**B**) Cell viability was determined by MTT assay in VSMCs incubated with LysoPC for 24 h (*n* = 8 in each group). (**C**) Cell viability was determined by MTT assay in VSMC incubated with 30 µM LysoPC plus acacetin for 24 h (*n* = 9 in each group). (**D**) Representative flow cytometry graphs of apoptosis in cells incubated with 30 µM LysoPC or LysoPC plus 3 µM acacetin for 24 h. (**E**) Data analysis of D (*n* = 8 in each group). (**F**) Representative Western blots of Bcl-2, Bax, and β-actin and (**G**) relative protein levels of Bcl-2 and Bax in VSMCs treated with LysoPC or LysoPC plus acacetin (*n* = 5 in each group). * *p* < 0.05, ** *p* < 0.01, *** *p* < 0.001, **** *p* < 0.0001, and ns *p* > 0.05.

As shown in Figure 1, acacetin showed significant cytoprotective effects against LysoPC-induced apoptosis in rat aortic VSMCs. Consistent with previous findings in human umbilical vein endothelial cells (HUVECs) [31], acacetin (0.3–3 μM) exhibited no intrinsic cytotoxicity in VSMCs across all tested concentrations (Figure 1A, *n* = 6). LysoPC treatment (10–60 μM) concentration-dependently reduced cellular viability (Figure 1B, *n* = 8). Pharmacological intervention with acacetin moderately ameliorated LysoPC (30 μM)-induced viability loss (Figure 1C, *n* = 9). Flow cytometric quantification revealed that LysoPC triggered an increase in apoptotic population, which was substantially attenuated by acacetin pretreatment (Figure 1D,E, *n* = 8). Mechanistically, Western blot analysis demonstrated that LysoPC exposure (30 μM, 24 h) significantly downregulated the anti-apoptotic protein Bcl-2 while upregulating pro-apoptotic Bax (Figure 1F,G, *n* = 5). Acacetin reversed these perturbations in a concentration-dependent manner. These findings show that acacetin confers cytoprotection against LysoPC-induced VSMC apoptosis through increasing Bcl-2 protein expression and decreasing Bax protein expression, suggesting its potential therapeutic utility in stabilizing atherosclerotic plaques through VSMC preservation.

### 3.2. Acacetin Suppresses LysoPC-Induced ROS Over-Production

**Figure 2:**
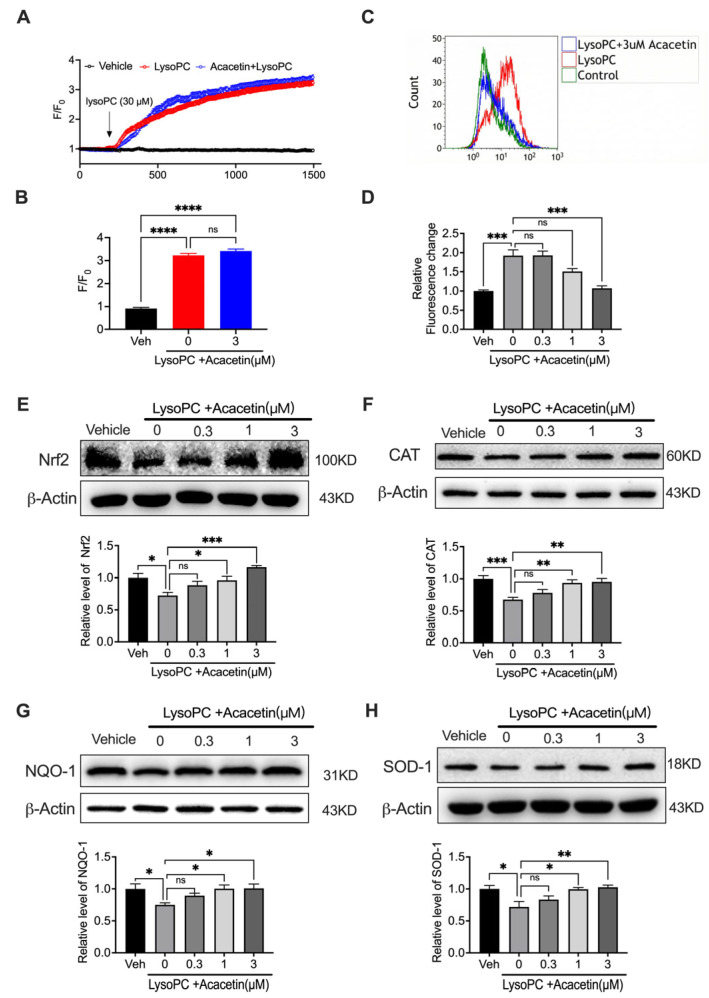
Acacetin restrained LysoPC-induced oxidative stress in VSMCs. (**A**) Representative Ca^2+^_i_ traces were recorded in cells treated with vehicle, 30 µM LysoPC, or 3 µM acacetin plus LysoPC for 24 h. (**B**) Mean values in cells treated as in A (*n* = 4 in each group). (**C**) Representative flow cytometry graph of ROS and data analysis ((**D**), *n* = 4 in each group) of intracellular ROS in cells treated with 30 µM LysoPC or 3 µM acacetin plus LysoPC. (**E**) Representative Western blots and relative level of Nrf2 in cells treated with 30 µM LysoPC and acacetin (0.3–3 μM) (*n* = 5 in each group). (**F**) Representative Western blots and relative level of CAT in cells treated with 30 µM LysoPC and acacetin (0.3–3 μM) (*n* = 6 in each group). (**G**) Representative Western blots and relative level of NQO-1 in cells treated with 30 µM LysoPC and acacetin (0.3–3 μM) (*n* = 5 in each group). (**H**) Representative Western blots and relative level of SOD-1 in cells treated with 30 µM LysoPC and acacetin (0.3–3 μM) (*n* = 4 in each group). * *p* < 0.05, ** *p* < 0.01, *** *p* < 0.001, **** *p* < 0.0001, and ns *p* > 0.05.

Building upon our prior demonstration of TRPC1/3-mediated Ca^2+^ dysregulation in LysoPC-induced human coronary SMC apoptosis [12], we investigated whether acacetin’s anti-apoptotic effects involved calcium modulation. Confocal imaging revealed 30 μM LysoPC triggered an increase in cytosolic Ca^2+^, which persisted despite 3 μM acacetin co-treatment (Figure 2A,B, *n* = 4), effectively excluding Ca^2+^-dependent mechanisms in acacetin’s cytoprotection.

We subsequently investigated whether ROS-mediated signal pathway involved the cytoprotection of acacetin using DCFH-DA flow cytometry. LysoPC (30 μM) induced a ROS surge, which was concentration-dependently mitigated by acacetin (Figure 2C,D, *n* = 4). The ROS attenuation paralleled the anti-apoptotic efficacy of acacetin, suggesting that redox regulation is the primary protective mechanism. Mechanistic investigation of the Nrf2 antioxidant pathway demonstrated LysoPC significantly suppressed key mediators: Nrf2, catalase, NQO1, and SOD1. Acacetin (0.3–3 μM) concentration-dependently restored their expression (Figure 2E–H, *n* = 4–6). Strikingly, acacetin at 3 μM normalized Nrf2 nuclear translocation, confirming pathway activation. These data establish that acacetin counteracts LysoPC-induced VSMC apoptosis primarily through Nrf2-driven transcriptional reactivation of antioxidant response, effectively neutralizing oxidative stress independent of Ca^2+^ signaling pathways.

### 3.3. Acacetin Orchestrates Nrf2 Nuclear Translocation via p62/Keap1 Axis

**Figure 3:**
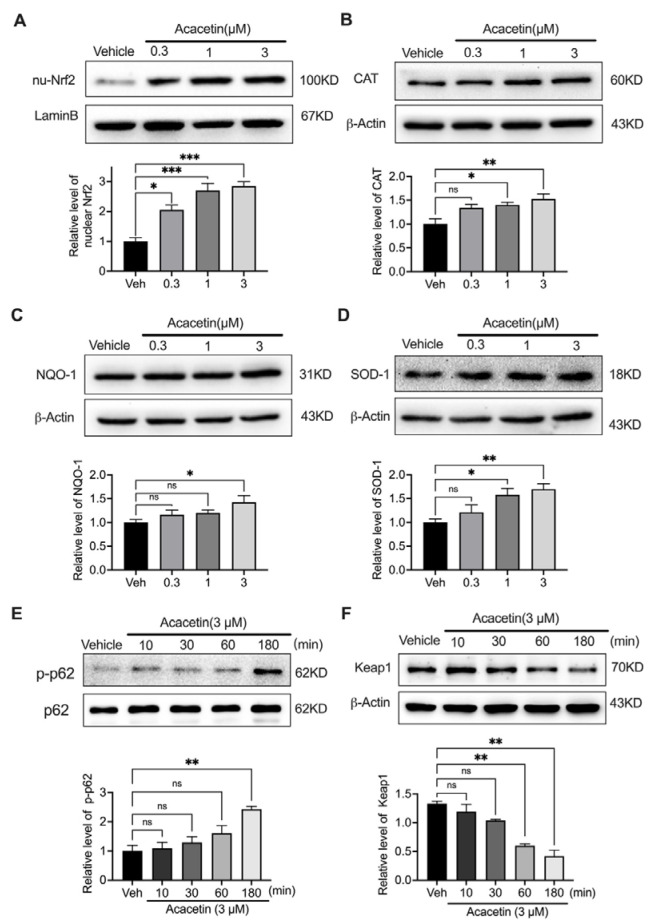
Acacetin regulated the Nrf2/p62/Keap1 signaling pathway in VSMCs. (**A**) Representative Western blots and relative level of nuclear Nrf2 in cells treated with vehicle or acacetin (0.3, 1, 3 μM) for 24 h (*n* = 4 in each group). (**B**) Representative Western blots and relative level of CAT in cells treated as in A (*n* = 6 in each group). (**C**) Representative Western blots and relative level of NQO-1 in cells treated as in A (*n* = 5 in each group). (**D**) Representative Western blots and relative level of SOD-1 in cells treated as in A (*n* = 5 in each group). (**E**) Representative Western blots and relative level of phosphorylated p62 in cells treated with vehicle or 3 µM acacetin for 10, 30, 60, and 180 min (*n* = 4 in each group). (**F**) Representative Western blots and relative level of Keap1 in cells treated as in E (*n* = 4 in each group). * *p* < 0.05, ** *p* < 0.01, *** *p* < 0.001, and ns *p* > 0.05.

The Nrf2-Keap1-p62 regulatory triad constitutes a pivotal oxidative stress response system [38]. Under basal conditions, Nrf2 forms a complex with Keap1 in the cytoplasm, while p62 phosphorylation at Ser349 facilitates competitive Keap1 binding, enabling Nrf2 liberation and nuclear translocation to activate antioxidant genes [39,40]. Western blot analysis revealed acacetin concentration-dependently enhanced nuclear Nrf2 accumulation (Figure 3A, *n* = 4), concomitant with upregulated expression of downstream effectors CAT, NQO1, and SOD1 (Figure 3B–D, *n* = 5–6). Temporal analysis demonstrated that 3 μM acacetin progressively increased p62 phosphorylation (Ser349) while reducing Keap1 levels (Figure 3E,F, *n* = 4). These findings present a novel mechanism whereby acacetin promotes Nrf2 nuclear shuttling through coordinated p62 activation and Keap1 suppression.

### 3.4. Genetic Ablation of Nrf2 Nullifies Cytoprotective Effects of Acacetin

**Figure 4:**
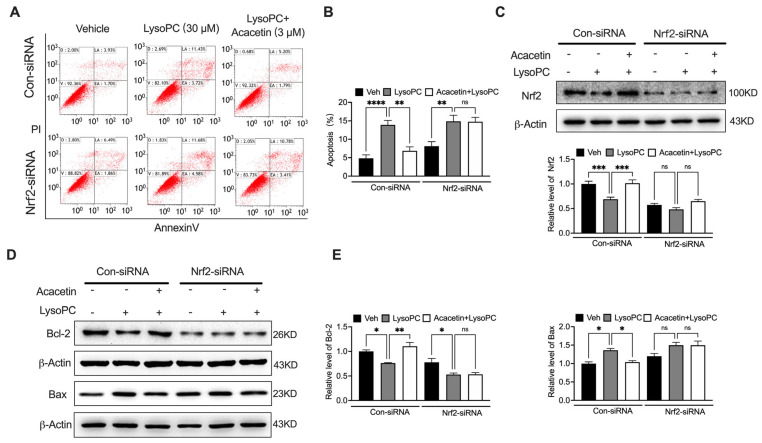
Silence of Nrf2 abolished the inhibition of acacetin on LysoPC-induced apoptosis in VSMCs. (**A**) Representative flow cytometry graphs of apoptosis in VSMCs transfected with control siRNA or Nrf2 siRNA (for 48 h) and treated with LysoPC or 3 μM acacetin plus LysoPC for 24 h. (**B**) Analysis of apoptosis in cells treated as in A (*n* = 8). (**C**) Representative Western blots and relative level of Nrf2 in cells treated as in A (*n* = 5 in each group). (**D**) Representative Western blots and (**E**) relative level of Bcl2 (*n* = 4 in each group) and Bax (*n* = 6 in each group) in VSMCs treated as in A. * *p* < 0.05, ** *p* < 0.01, *** *p* < 0.001, **** *p* < 0.0001, and ns *p* > 0.05.

**Figure 5:**
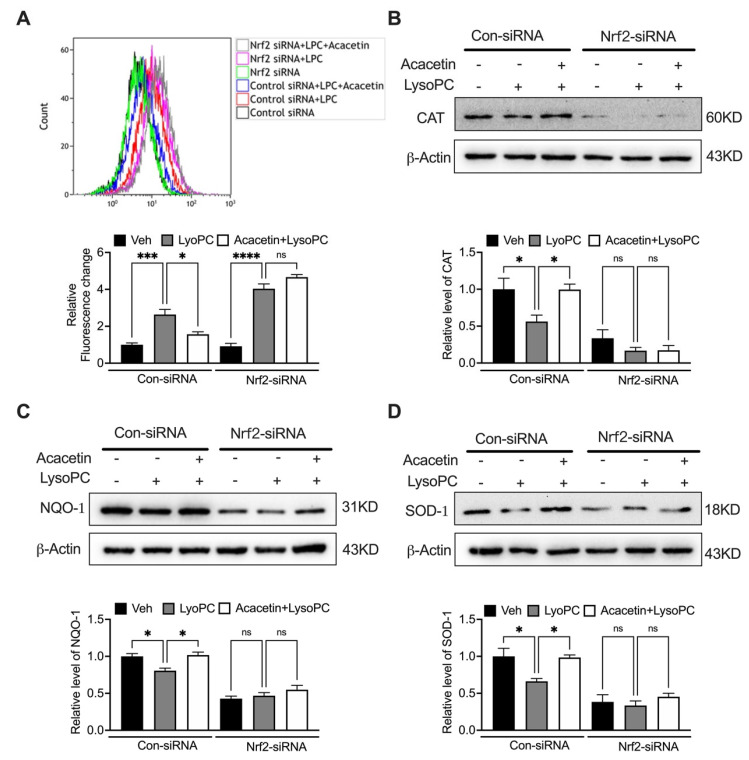
Silence of Nrf2 abolished acacetin-suppressed ROS production in VSMCs. (**A**) Representative flow cytometry graphs of intracellular ROS and relative levels of ROS in cells transfected with control siRNA or Nrf2 siRNA (for 48 h) and treated with LysoPC or 3 μM acacetin plus LysoPC for 24 h (*n* = 4 in each group). (**B**) Representative Western blots and relative level of CAT in cells treated as in A (*n* = 4 in each group). (**C**) Representative Western blots and relative level of NQO-1 in cells treated as in A (*n* = 5 in each group). (**D**) Representative Western blots and relative level of SOD-1 in cells treated as in A (*n* = 4 in each group). * *p* < 0.05, *** *p* < 0.001, **** *p* < 0.0001, and ns *p* > 0.05.

SiRNA Silence of Nrf2 completely abolished acacetin-mediated protection. In scramble controls, acacetin (3 μM) reversed LysoPC-induced viability reduction and apoptosis, while Nrf2-silenced cells remained unresponsive (Figure 4A–C, *n* = 5–8). Similarly, the modulation of Bcl-2 increase and Bax decrease by acacetin was abrogated upon Nrf2 depletion (Figure 4D,E, *n* = 4–6). Consistent with apoptotic regulation, the antioxidant capacity of acacetin proved to be Nrf2-dependent. While acacetin attenuated LysoPC-induced ROS surge in control cells, this effect was absent in Nrf2-deficient VSMCs (Figure 5A, *n* = 4). The rescue of antioxidant enzymes by acacetin—CAT, NQO1, SOD1—strictly required Nrf2 expression (Figure 5B–D, *n* = 4–5). These data unequivocally position Nrf2 as the central mediator of acacetin’s pleiotropic protective effects.

### 3.5. Sirt1 Governs Acacetin-Induced Nrf2 Activation in VSMCs

**Figure 6:**
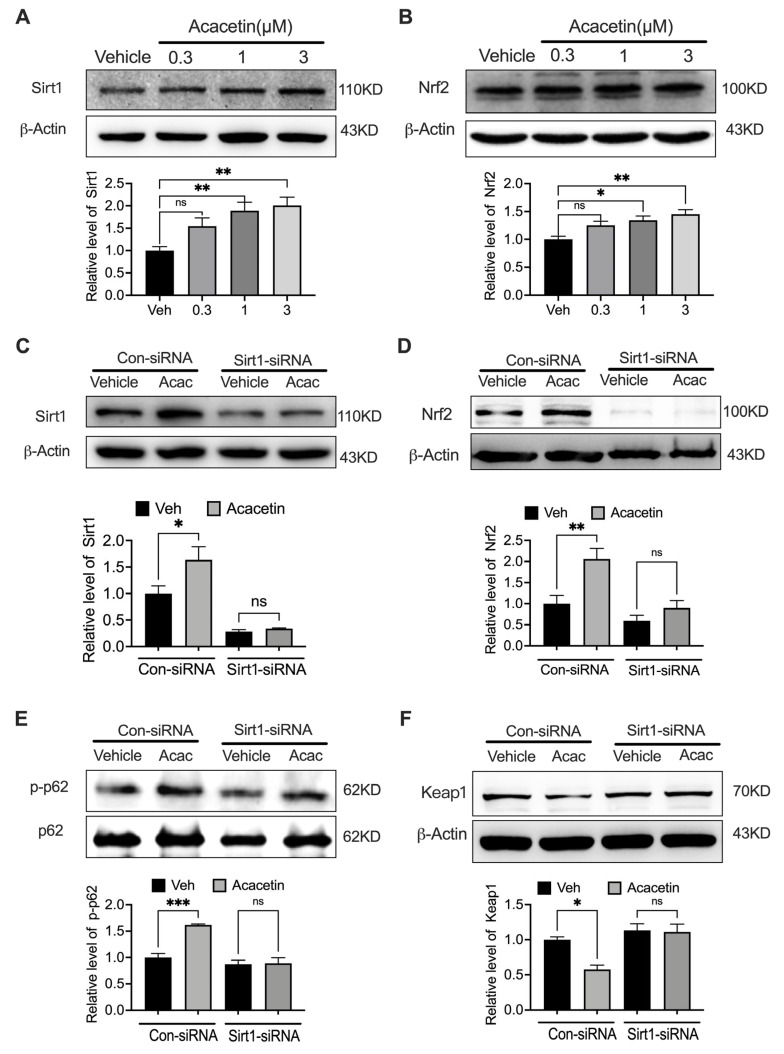
Sirt1 is the upstream target of acacetin-induced Nrf2 activation in VSMCs. (**A**) Representative Western blots and relative level of nuclear Sirt1 in cells treated with acacetin (0.3–3 μM) for 24 h (*n* = 6 in each group). (**B**) Representative Western blots and relative level of Nrf2 in cells treated with acacetin (0.3–3 μM) for 24 h (*n* = 5 in each group). (**C**) Representative Western blots and relative level of Sirt1 in cells transfected with control siRNA or Sirt1 siRNA (for 48 h), then treated with vehicle (Veh) or 3 μM acacetin (24 h) (*n* = 4 in each group). (**D**) Representative Western blots and relative level of Nrf2 in cells treated as in C (*n* = 5 in each group). (**E**) Representative Western blots and relative level of phosphorylated p62 in cells treated as in C (*n* = 4 in each group). (**F**) Representative Western blots and relative level of Keap1 in cells treated as in C (*n* = 4 in each group). * *p* < 0.05, ** *p* < 0.01, *** *p* < 0.001, and ns *p* > 0.05.

Building on our prior demonstrations of Sirt1-dependent Nrf2 regulation in cardiovascular cells [27,28,30,31], we identified a conserved mechanism in VSMCs. Acacetin concentration-dependently upregulated Sirt1 and Nrf2 protein levels (Figure 6A,B, *n* = 5–6). Sirt1 silencing prevented acacetin-induced: Sirt1/Nrf2 protein elevation (Figure 6C,D, *n* = 4–5), p62 phosphorylation (Ser349, Figure 6E, *n* = 4), and Keap1 downregulation (Figure 6F, *n* = 4). This comprehensive analysis establishes Sirt1 as the upstream regulator of acacetin’s antioxidant signaling through the Nrf2/p62/Keap1 axis, revealing therapeutic targeting potential of acacetin in vascular pathologies.

### 3.6. Acacetin Rescues Sirt1-Mediated Vascular Homeostasis in Atherosclerotic ApoE^−/−^ Mice

**Figure 7:**
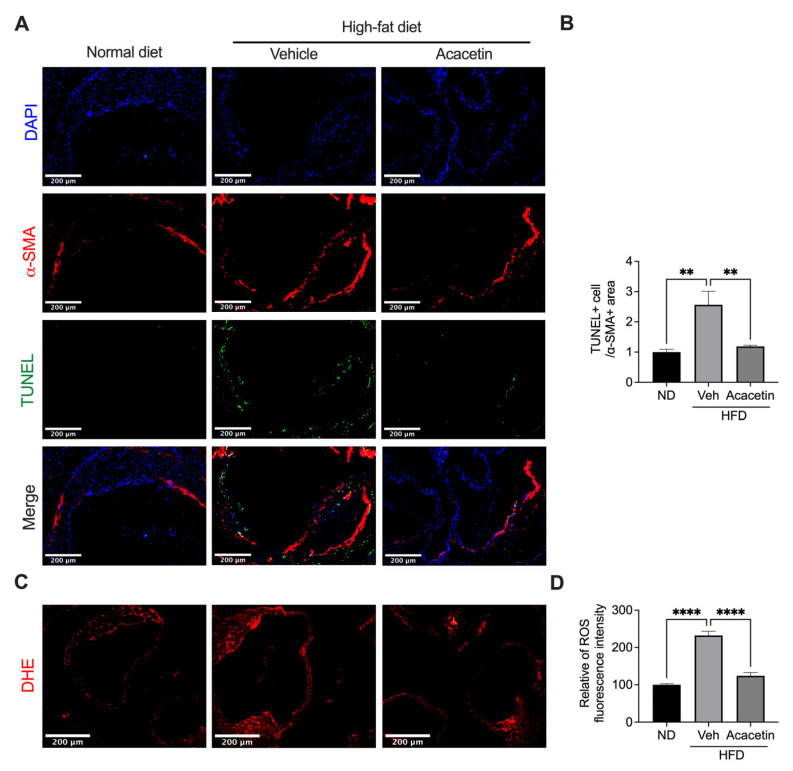
Acacetin downregulated apoptosis and ROS production in aortic tissues of HFD ApoE^−/−^ mice. (**A**) Representative images of α-SMA and TUNEL (scale bar = 200 μm) and (**B**) relative level of apoptotic cells in α-SMA-positive cells in aortic tissues of ApoE^−/−^ mice (*n* = 7 in each group). (**C**) Representative images of ROS fluorescence stain (scale bar = 200 μm) and (**D**) relative level of ROS in aortic tissues of ApoE^−/−^ mice (*n* = 9 in each group). ** *p* < 0.01, **** *p =* <0.0001.

**Figure 8:**
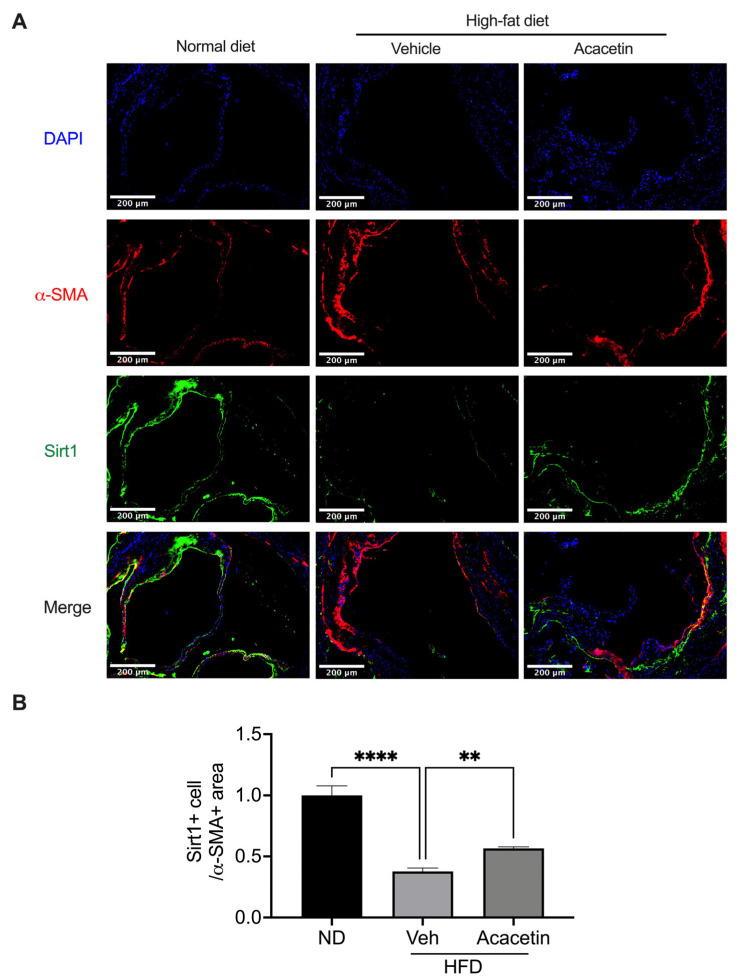
Acacetin rescued the downregulated expression of Sirt1 in HFD ApoE^−/−^ mice. (**A**) Representative fluorescent images of α-SMA and Sirt1 (scale bar = 200 μm) and (**B**) relative level of Sirt1 in α-SMA-positive cells in aortic tissues from HFD ApoE^−/−^ mice (*n* = 7 in each group). ** *p* < 0.01, **** *p* < 0.0001.

Building upon our prior demonstration of acacetin’s potent anti-atherogenic efficacy in HFD-fed ApoE^−/−^ mice [32], we herein demonstrate its cellular protective mechanisms within aortic tissues. Histomorphometric analysis revealed HFD-induced profound VSMC apoptosis and oxidative stress, which were markedly attenuated by chronic acacetin treatment (15 mg/kg/day, 12 weeks, Figure 7A–D, *n* = 7–9). This in vivo protection mirrored our in vitro findings with LysoPC-challenged VSMCs, validating the pathophysiological relevance of our mechanistic model. Mechanistically, immunoblot quantification demonstrated HFD-driven Sirt1 downregulation in aortic lysates, which was significantly rescued by acacetin (Figure 8A,B, *n* = 7). These translational findings substantiate that acacetin’s vascular protective effects in complex atherosclerotic milieus operate through conserved Sirt1 activation mechanisms, bridging cellular-level discoveries with whole-organism pathophysiology.

## 4. Discussion

LysoPC induced VSMC apoptosis and inflammation mediated by concurrent calcium dysregulation and ROS overproduction [12], while the results from the present study support the notion that apoptosis and inflammation induced by LysoPC are effectively countered by acacetin via restoring the damaged redox homeostasis rather than calcium homeostasis modulation. This study elucidates a vasoprotective signal axis of acacetin targeting Sirt1/Nrf2/p62 pathway in VSMCs. Acacetin orchestrates antioxidant response through sequential Sirt1 activation → Nrf2 nuclear translocation → transcriptional upregulation of NQO-1/SOD1, establishing a multi-tiered protective cascade in VSMCs.

Our previous studies have demonstrated that acacetin has significant cardiovascular protection in different in vitro and in vivo models, in which the Sirt1-mediated signaling cascade is involved [14]. In addition to reversing hypoxia pulmonary hypertension via inhibiting pulmonary artery VSMC hyper-proliferation via Sirt1-HMGB1 signal regulation [33], acacetin inhibits myocardial hypertrophy through the Sirt1/AMPK/PGC-1α pathway [28], attenuates aging heart failure through Sirt1-mediated activation of Sirt6/AMPK signaling pathway [30], improves doxorubicin-induced cardiomyopathy through the Sirt1/AMPK/Nrf2 signaling pathway [27] and alleviates endothelial cell damage through the Sirt1/Sirt3/AMPK mitochondrial axis and MsrA-Nrf2/Keap1 cascade [32]. In this study, we demonstrate the novel pharmacological effect that the protection of acacetin against LysoPC-induced VSMC injury was mediated by activating Sirt1/Nrf2 molecules followed by increasing p62 phosphorylation, which is different from those in other cell types.

Therapeutic validation in HFD-fed ApoE^−/−^ mice revealed the capacity of acacetin to rescue Sirt1 expression, concomitantly reducing vascular oxidative stress and apoptosis. This translational consistency bridges cellular mechanisms with pathophysiological outcomes, positioning acacetin as a pleiotropic vascular modulator and expanding its established pharmacopeia—spanning atrial fibrillation management through ion channel blockade (atrial-selective I_Kur_ channel inhibition) [21] and metabolic regulation (hepatic DPP4 inhibition) [41]. Although suppression of LysoPC-induced Ca^2+^ influx is important for the VSMCs’ protection [11,12], LysoPC-induced Ca^2+^ influx was not effectively prevented in VSMCs with acacetin, indicating that acacetin plays a critical role in vascular protection by maintaining redox homeostasis involved in Sirt1/Nrf2 pathway, but not by Ca^2+^ modulation. Our findings augment the therapeutic portfolio of acacetin. This VSMC-focused discovery completes acacetin’s cellular defense triad (cardiomyocyte-endothelial-VSMC), underscoring its unique capacity to target multiple atherosclerotic components through unified redox/Nrf2 signaling.

The Nrf2-Keap1-p62 axis constitutes an evolutionarily conserved cytoprotective system, where Nrf2 serves as a central regulator of cellular redox homeostasis and inflammatory responses through transcriptional control of antioxidant enzymes (NQO1, CAT, SOD) and detoxification proteins [42]. Under physiological conditions, Keap1 anchors Nrf2 in the cytoplasm for proteasomal degradation, while stress-induced p62 phosphorylation at Ser349 disrupts this complex, enabling Nrf2 nuclear translocation and subsequent activation of antioxidant response element (ARE)-driven genes [39,43]. Our findings demonstrate that acacetin (1 and 3 μM) protects against LysoPC-induced VSMC injury by enhancing p62 phosphorylation to competitively bind Keap1 and suppressing Keap1 expression, collectively promoting nuclear Nrf2 accumulation and upregulating cytoprotective enzymes (NQO1 and SOD1). Genetic silence of Nrf2 completely abolished these effects, corroborating its indispensable role—a phenomenon consistent with our previous observations in endothelial cells and cardiomyocytes [27,44].

Crucially, the Nrf2 activation is governed by upstream Sirt1, an NAD^+^-dependent deacetylase that integrates metabolic sensing with stress adaptation through targets spanning PGC-1α to NF-κB [45]. Activating Sirt1/Nrf2/p62 signaling may inhibit oxidative stress, inflammation, and autophagy through mediating autophagy-dependent ferroptosis [46]. Clinically relevant Sirt1 deficiency evidenced by lower serum levels in coronary atherosclerosis patients [47] was recapitulated in LysoPC-challenged VSMCs and HFD-fed ApoE^−/−^ aortic tissues, both rescued by acacetin treatment. Sirt1 silencing abolished acacetin-mediated Nrf2/p62 activation, mechanistically linking Sirt1 to the Nrf2 pathway, a regulatory axis we previously characterized in cardiac [28] and endothelial [31] systems.

Although these results are encouraging, there are limitations that should be noted. Firstly, the specific upstream kinases for p62 phosphorylation, the acetylation profiles of Nrf2 and p62, how exactly they are regulated by Sirt1, and the impact of acacetin on these proteins need to be further clarified. Second, this study relied mainly on cellular tools like siRNA to demonstrate pathway associations; therefore, future studies using Sirt1 conditional knockout mice are needed for in vivo verification. Finally, whether the Sirt1/Nrf2/p62 axis operates in the same manner in advanced human plaques accompanied by severe calcification and macrophage infiltration, as well as the clinical targeted delivery strategies for acacetin, remain to be studied.

## 5. Conclusions

These findings position acacetin as a unique modulator, concurrently addressing oxidative stress (via Sirt1/Nrf2/p62) and metabolic dysregulation (through Sirt1’s deacetylation network). With demonstrated efficacy in reducing VSMC apoptosis and ROS production in preclinical models, acacetin emerges as a promising therapeutic drug candidate for atherosclerotic plaque stabilization through multimodal vascular protection.

## Data Availability

The original contributions presented in this study are included in the article. Further inquiries can be directed to the corresponding authors.

## References

[1] Goncalves I., Edsfeldt A., Ko N.Y., Grufman H., Berg K., Bjorkbacka H., Nitulescu M., Persson A., Nilsson M., Prehn C. (2012). Evidence supporting a key role of Lp-PLA2-generated lysophosphatidylcholine in human atherosclerotic plaque inflammation. Arterioscler. Thromb. Vasc. Biol..

[2] Dohi T., Miyauchi K., Ohkawa R., Nakamura K., Kurano M., Kishimoto T., Yanagisawa N., Ogita M., Miyazaki T., Nishino A. (2013). Increased lysophosphatidic acid levels in culprit coronary arteries of patients with acute coronary syndrome. Atherosclerosis.

[3] Law S.H., Chan M.L., Marathe G.K., Parveen F., Chen C.H., Ke L.Y. (2019). An Updated Review of Lysophosphatidylcholine Metabolism in Human Diseases. Int. J. Mol. Sci..

[4] Kume H., Harigane R., Rikimaru M. (2024). Involvement of Lysophospholipids in Pulmonary Vascular Functions and Diseases. Biomedicines.

[5] Corrêa R., Silva L.F.F., Ribeiro D.J.S., Almeida R.D.N., Santos I.O., Corrêa L.H., de Sant’Ana L.P., Assunção L.S., Bozza P.T., Magalhães K.G. (2019). Lysophosphatidylcholine Induces NLRP3 Inflammasome-Mediated Foam Cell Formation and Pyroptosis in Human Monocytes and Endothelial Cells. Front. Immunol..

[6] Matsumoto T., Kobayashi T., Kamata K. (2007). Role of lysophosphatidylcholine (LPC) in atherosclerosis. Curr. Med. Chem..

[7] Kurano M., Kano K., Dohi T., Matsumoto H., Igarashi K., Nishikawa M., Ohkawa R., Ikeda H., Miyauchi K., Daida H. (2017). Different origins of lysophospholipid mediators between coronary and peripheral arteries in acute coronary syndrome. J. Lipid Res..

[8] Kurano M., Suzuki A., Inoue A., Tokuhara Y., Kano K., Matsumoto H., Igarashi K., Ohkawa R., Nakamura K., Dohi T. (2015). Possible involvement of minor lysophospholipids in the increase in plasma lysophosphatidic acid in acute coronary syndrome. Arterioscler. Thromb. Vasc. Biol..

[9] Tanaka T., Ikeda K., Yamamoto Y., Iida H., Kikuchi H., Morita T., Yamasoba T., Nagai R., Nakajima T. (2011). Effects of serum amyloid a and lysophosphatidylcholine on intracellular calcium concentration in human coronary artery smooth muscle cells. Int. Heart J..

[10] Terasawa K., Nakajima T., Iida H., Iwasawa K., Oonuma H., Jo T., Morita T., Nakamura F., Fujimori Y., Toyo-oka T. (2002). Nonselective cation currents regulate membrane potential of rabbit coronary arterial cell: Modulation by lysophosphatidylcholine. Circulation.

[11] Kim M.Y., Liang G.H., Kim J.A., Choi S.S., Choi S., Suh S.H. (2009). Oxidized Low-density Lipoprotein- and Lysophosphatidylcholine-induced Ca Mobilization in Human Endothelial Cells. Korean J. Physiol. Pharmacol..

[12] Wang Y., Wang Y., Li G.R. (2016). TRPC1/TRPC3 channels mediate lysophosphatidylcholine-induced apoptosis in cultured human coronary artery smooth muscles cells. Oncotarget.

[13] Chang W., Wu Q.Q., Xiao Y., Jiang X.H., Yuan Y., Zeng X.F., Tang Q.Z. (2017). Acacetin protects against cardiac remodeling after myocardial infarction by mediating MAPK and PI3K/Akt signal pathway. J. Pharmacol. Sci..

[14] Wang S.Y., Wang Y.J., Dong M.Q., Li G.R. (2024). Acacetin is a Promising Drug Candidate for Cardiovascular Diseases. Am. J. Chin. Med..

[15] Chien S.T., Lin S.S., Wang C.K., Lee Y.B., Chen K.S., Fong Y., Shih Y.W. (2011). Acacetin inhibits the invasion and migration of human non-small cell lung cancer A549 cells by suppressing the p38alpha MAPK signaling pathway. Mol. Cell Biochem..

[16] Kim C.D., Cha J.D., Li S., Cha I.H. (2015). The mechanism of acacetin-induced apoptosis on oral squamous cell carcinoma. Arch. Oral. Biol..

[17] Wang S., Lin B., Liu W., Wei G., Li Z., Yu N., Xue X., Ji G. (2020). Acacetin Induces Apoptosis in Human Osteosarcoma Cells by Modulation of ROS/JNK Activation. Drug Des. Devel Ther..

[18] Tian M., Tang Y., Huang T., Liu Y., Pan Y. (2021). Amelioration of human peritoneal mesothelial cell co-culture-evoked malignant potential of ovarian cancer cells by acacetin involves LPA release-activated RAGE-PI3K/AKT signaling. Cell Mol. Biol. Lett..

[19] Yun S., Lee Y.J., Choi J., Kim N.D., Han D.C., Kwon B.M. (2021). Acacetin Inhibits the Growth of STAT3-Activated DU145 Prostate Cancer Cells by Directly Binding to Signal Transducer and Activator of Transcription 3 (STAT3). Molecules.

[20] Zhang G., Li Z., Dong J., Zhou W., Zhang Z., Que Z., Zhu X., Xu Y., Cao N., Zhao A. (2022). Acacetin inhibits invasion, migration and TGF-beta1-induced EMT of gastric cancer cells through the PI3K/Akt/Snail pathway. BMC Complement. Med. Ther..

[21] Li G.R., Wang H.B., Qin G.W., Jin M.W., Tang Q., Sun H.Y., Du X.L., Deng X.L., Zhang X.H., Chen J.B. (2008). Acacetin, a natural flavone, selectively inhibits human atrial repolarization potassium currents and prevents atrial fibrillation in dogs. Circulation.

[22] Liu H., Wang Y.J., Yang L., Zhou M., Jin M.W., Xiao G.S., Wang Y., Sun H.Y., Li G.R. (2016). Synthesis of a highly water-soluble acacetin prodrug for treating experimental atrial fibrillation in beagle dogs. Sci. Rep..

[23] Wu H.J., Sun H.Y., Wu W., Zhang Y.H., Qin G.W., Li G.R. (2013). Properties and molecular determinants of the natural flavone acacetin for blocking hKv4.3 channels. PLoS ONE.

[24] Wu H.J., Wu W., Sun H.Y., Qin G.W., Wang H.B., Wang P., Yalamanchili H.K., Wang J., Tse H.F., Lau C.P. (2011). Acacetin causes a frequency- and use-dependent blockade of hKv1.5 channels by binding to the S6 domain. J. Mol. Cell Cardiol..

[25] Liu H., Yang L., Wu H.J., Chen K.H., Lin F., Li G., Sun H.Y., Xiao G.S., Wang Y., Li G.R. (2016). Water-soluble acacetin prodrug confers significant cardioprotection against ischemia/reperfusion injury. Sci. Rep..

[26] Wu W.Y., Li Y.D., Cui Y.K., Wu C., Hong Y.X., Li G., Wu Y., Jie L.J., Wang Y., Li G.R. (2018). The Natural Flavone Acacetin Confers Cardiomyocyte Protection Against Hypoxia/Reoxygenation Injury via AMPK-Mediated Activation of Nrf2 Signaling Pathway. Front. Pharmacol..

[27] Wu W.Y., Cui Y.K., Hong Y.X., Li Y.D., Wu Y., Li G., Li G.R., Wang Y. (2020). Doxorubicin cardiomyopathy is ameliorated by acacetin via Sirt1-mediated activation of AMPK/Nrf2 signal molecules. J. Cell Mol. Med..

[28] Cui Y.K., Hong Y.X., Wu W.Y., Han W.M., Wu Y., Wu C., Li G.R., Wang Y. (2022). Acacetin ameliorates cardiac hypertrophy by activating Sirt1/AMPK/PGC-1alpha pathway. Eur. J. Pharmacol..

[29] Song F., Mao Y.J., Hu Y., Zhao S.S., Wang R., Wu W.Y., Li G.R., Wang Y., Li G. (2022). Acacetin attenuates diabetes-induced cardiomyopathy by inhibiting oxidative stress and energy metabolism via PPAR-alpha/AMPK pathway. Eur. J. Pharmacol..

[30] Hong Y.X., Wu W.Y., Song F., Wu C., Li G.R., Wang Y. (2021). Cardiac senescence is alleviated by the natural flavone acacetin via enhancing mitophagy. Aging.

[31] Han W.M., Chen X.C., Li G.R., Wang Y. (2020). Acacetin Protects Against High Glucose-Induced Endothelial Cells Injury by Preserving Mitochondrial Function via Activating Sirt1/Sirt3/AMPK Signals. Front. Pharmacol..

[32] Wu Y., Song F., Li Y., Li J., Cui Y., Hong Y., Han W., Wu W., Lakhani I., Li G. (2021). Acacetin exerts antioxidant potential against atherosclerosis through Nrf2 pathway in apoE^−/−^ Mice. J. Cell Mol. Med..

[33] Wang H., Peng L.J., Lu W., Li G.R., Zhao P.T., Lv X., Dong M.Q., Liu M.L. (2025). Acacetin reverses hypoxic pulmonary hypertension by inhibiting hypoxia-induced proliferation of pulmonary artery smooth muscle cells via SIRT1-HMGB1 pathway. Eur. J. Pharmacol..

[34] Su X.L., Wang Y., Zhang W., Zhao L.M., Li G.R., Deng X.L. (2011). Insulin-mediated upregulation of K(Ca)3.1 channels promotes cell migration and proliferation in rat vascular smooth muscle. J. Mol. Cell Cardiol..

[35] Che H., Li G., Sun H.Y., Xiao G.S., Wang Y., Li G.R. (2015). Roles of store-operated Ca^2+^ channels in regulating cell cycling and migration of human cardiac c-kit^+^ progenitor cells. Am. J. Physiol. Heart Circ. Physiol..

[36] Li G., Che H., Wu W.Y., Jie L.J., Xiao G.S., Wang Y., Li G.R. (2018). Bradykinin-mediated Ca^2+^ signalling regulates cell growth and mobility in human cardiac c-Kit^+^ progenitor cells. J. Cell Mol. Med..

[37] Chen J.B., Tao R., Sun H.Y., Tse H.F., Lau C.P., Li G.R. (2010). Multiple Ca^2+^ signaling pathways regulate intracellular Ca^2+^ activity in human cardiac fibroblasts. J. Cell Physiol..

[38] Vomund S., Schafer A., Parnham M.J., Brune B., von Knethen A. (2017). Nrf2, the Master Regulator of Anti-Oxidative Responses. Int. J. Mol. Sci..

[39] Komatsu M., Kurokawa H., Waguri S., Taguchi K., Kobayashi A., Ichimura Y., Sou Y.S., Ueno I., Sakamoto A., Tong K.I. (2010). The selective autophagy substrate p62 activates the stress responsive transcription factor Nrf2 through inactivation of Keap1. Nat. Cell Biol..

[40] Kageyama S., Saito T., Obata M., Koide R.H., Ichimura Y., Komatsu M. (2018). Negative Regulation of the Keap1-Nrf2 Pathway by a p62/Sqstm1 Splicing Variant. Mol. Cell Biol..

[41] Liou C.J., Wu S.J., Shen S.C., Chen L.C., Chen Y.L., Huang W.C. (2022). Acacetin Protects against Non-Alcoholic Fatty Liver Disease by Regulating Lipid Accumulation and Inflammation in Mice. Int. J. Mol. Sci..

[42] Kobayashi M., Yamamoto M. (2005). Molecular mechanisms activating the Nrf2-Keap1 pathway of antioxidant gene regulation. Antioxid. Redox Signal..

[43] Dayalan Naidu S., Dinkova-Kostova A.T. (2020). KEAP1, a cysteine-based sensor and a drug target for the prevention and treatment of chronic disease. Open Biol..

[44] Wu D., Wang Y., Zhang H., Du M., Li T. (2018). Acacetin attenuates mice endotoxin-induced acute lung injury via augmentation of heme oxygenase-1 activity. Inflammopharmacology.

[45] Iside C., Scafuro M., Nebbioso A., Altucci L. (2020). SIRT1 Activation by Natural Phytochemicals: An Overview. Front. Pharmacol..

[46] Han Q., Gu Y., Qian Y. (2025). Study on the mechanism of activating SIRT1/Nrf2/p62 pathway to mediate autophagy-dependent ferroptosis to promote healing of diabetic foot ulcers. Naunyn Schmiedebergs Arch. Pharmacol..

[47] He X., Zheng J., Liu C. (2019). Low serum level of sirtuin 1 predicts coronary atherosclerosis plaques during computed tomography angiography among an asymptomatic cohort. Coron. Artery Dis..

